# Conflict and conflict resolution in the major transitions

**DOI:** 10.1098/rspb.2023.1420

**Published:** 2023-10-11

**Authors:** Andrew F. G. Bourke

**Affiliations:** School of Biological Sciences, University of East Anglia, Norwich Research Park, Norwich NR4 7TJ, UK

**Keywords:** conflict, eusociality, inclusive fitness, individuality, major transition, organismality

## Abstract

Conflict and conflict resolution have been argued to be fundamental to the major transitions in evolution. These were key events in life's history in which previously independently living individuals cooperatively formed a higher-level individual, such as a multicellular organism or eusocial colony. Conflict has its central role because, to proceed stably, the evolution of individuality in each major transition required within-individual conflict to be held in check. This review revisits the role of conflict and conflict resolution in the major transitions, addressing recent work arguing for a minor role. Inclusive fitness logic suggests that differences between the kin structures of clones and sexual families support the absence of conflict at the origin of multicellularity but, by contrast, suggest that key conflicts existed at the origin of eusociality. A principal example is conflict over replacing the founding queen (queen replacement). Following the origin of each transition, conflict remained important, because within-individual conflict potentially disrupts the attainment of maximal individuality (organismality) in the system. The conclusion is that conflict remains central to understanding the major transitions, essentially because conflict arises from differences in inclusive fitness optima while conflict resolution can help the system attain a high degree of coincidence of inclusive fitness interests.

## 1. Introduction

### (a) Conflict and conflict resolution

Conflict is an integral feature of social evolution. It has long been recognized that, when different parties within social groups have differing inclusive fitness optima, they are potentially in conflict [1–3]. (Here, parties are individuals or sets of individuals (e.g. kin) within social groups, an inclusive fitness optimum is the value of a given trait that maximizes inclusive fitness for a given party, and conflict refers to an evolutionary conflict of interest.) Differing inclusive fitness optima arise whenever group kin structure falls short of clonality, because different sets of kin within the group then have unequal genetic relatednesses to the group's potential offspring [1–5]. As a corollary, full cooperation within social groups is expected only when the parties, whatever the group's kin structure, have completely overlapping inclusive fitness optima [e.g. 6,7]. Conflict as a term is also used in other contexts. For example, in a multicellular organism, a trade-off may exist between somatic and germline cells over the optimal level of investment between organismal survival and reproduction, and this trade-off is sometimes referred to as a conflict. However, given a multicellular organism's clonal kin structure (and ignoring post-zygotic mutations), somatic and germline cells have a complete coincidence of inclusive fitness interests, including with respect to the optimal level of investment between organismal survival and reproduction. So, from an inclusive fitness standpoint, this case, though a trade-off, is not a conflict [5,8]. In the current review, conflict will refer only to situations in which, within social groups, different parties exhibit unequal inclusive fitness optima.

When a potential conflict has physical expression, e.g. fighting between group-mates, it is said to become an actual conflict [2]. Although kin structure dictates whether potential conflict is present within a social group, such conflict does not inevitably become actual because a number of constraints may prevent this from happening [2,3,9]. For example, in eusocial Hymenoptera (ants, bees and wasps with a worker caste), potential conflict over queen rearing stemming from colonies under a multiply-mated queen containing a mixture of full- or half-sisters fails to become actual because workers lack effective within-colony kin discrimination. Actual conflict is prevented by an informational constraint [2,3,9]. Moreover, if actual conflict has arisen, an important modifier of it is conflict resolution [3,9]. In the current context, this is defined as any process that, over evolutionary time, leads to the costs of actual conflict being reduced, with costs being measured in terms of group productivity [3].

### (b) Major transitions in evolution

The major transitions in evolution are events in the history of life on the Earth in which previously independently living individuals cooperatively form a higher-level group representing a new level of individuality [9–17]. In this context, in line with previous definitions [9,10,13], an individual is ‘a stable, physically discrete entity composed of interdependent parts acting in a coordinated manner to achieve common reproductive goals, and typified by attenuated within-individual conflict’ [18, p. 62]. The canonical examples of major transitions to individuality are the evolution of the eukaryotic cell (from a non-eukaryotic unicell engulfing another in endosymbiosis), the evolution of multicellularity (from eukaryotic unicells) and the evolution of eusociality (from multicellular organisms) [9,11,17]. Queller [14] divided the major transitions into egalitarian ones (between unrelated partners) and fraternal ones (between related partners). The evolution of the eukaryotic cell is an egalitarian transition because it occurred between the ancestor of the nuclear genome and the ancestor of the unrelated organellar genome, and the evolution of multicellularity and eusociality are fraternal transitions because they occurred, respectively, between related cells and organisms [14].

While broader views of what constitutes a major transition have been developed [11,15,19], the current review focuses on major transitions involving the evolution of a higher level of individuality from a union of lower-level individuals [9,10,13,14,16,17]. This is because how and why living things occur in a nested hierarchy of individuality represent key questions in the study of evolution; and because, with this focus, the major transitions framework provides evolutionary biology's leading answer [9,16–18].

In [9], synthesizing and building on contributions of [10–14,20,21] and others, I argued that each fraternal major transition consists of the acquisition of social complexity in a process of positive feedback from an ancestry in simple societies (that themselves derived from solitary ancestors) and culminates in the evolution of individuality. I also suggested that, because (like all evolutionary change under natural selection) a major transition occurs generation by generation, it is best understood as a process. Nonetheless, each transition, viewed across the long reach of evolutionary time, represents a step-like change, and, when repeated within lineages, produces the set of nested entities (e.g. cells in organisms, organisms in eusocial colonies) that make up the biological hierarchy. Moreover, the overarching reason, based on first principles of inclusive fitness theory (box 1), for the evolution of individuality at any level is that this occurs when genes within the parties making up a social group experience a high level of coincidence of inclusive fitness interests [4,7,9,18].

Box 1. **Inclusive fitness theory, altruism and Hamilton's rule.**

Inclusive fitness theory [4] explains selection for social actions, including altruism. Altruism is the social action in which an actor pays a lifetime cost in direct fitness (offspring production) to increase the direct fitness of a recipient. Somatic cells in multicellular organisms, and workers in a eusocial colony, are altruists, because they sacrifice their own ability to produce offspring to help rear those of reproductive phenotypes (germline producing gametes and queen producing eggs, respectively). For current purposes, inclusive fitness theory can be summarized in Hamilton's rule [4]. This states, for the case of altruism in prospective helpers/workers via rearing sibs within a subsocial (parent–offspring) kin structure, that helping undergoes selection when *br*_sib_ > *cr*_off_, where *b* = number of additional sibs reared via helping, *c* = number of offspring lost via helping, *r*_sib_ = relatedness to sibs and *r*_off_ = relatedness to offspring.

### (c) Conflict and conflict resolution in relation to the major transitions

Conflict and conflict resolution have been argued to be fundamental components of the major transitions [9–11,13,14,16]. In [9], in line with the view of a major transition as a process, I proposed that each major transition could be usefully split into three, non-mutually exclusive stages—social group formation, social group maintenance and social group transformation. Social group formation refers to the initial union of previously separate individuals to form a social group. Social group maintenance refers to processes that keep a social group stable once it has formed. Lastly, social group transformation refers to processes of positive feedback by which simple social groups become complex, where the most complex social groups are those with the greatest degree of reproductive and non-reproductive division of labour and hence with the greatest degree of individuality. Social group transformation was therefore taken to be the stage in which the evolution of individuality, and hence the major transition, is completed [9].

Each of these three stages may, in principle, be affected by conflict and conflict resolution [9,11,14,16]. For example, in colonies of eusocial Hymenoptera, workers may produce male offspring from unfertilized eggs via haplodiploidy, which creates conflict over male parentage because members of each caste (queen or workers) are more closely related to their own caste's male offspring [1]. Worker policing (mutual prevention of one another's reproduction) helps to resolve this conflict and so to maintain group stability [3,22]. Worker policing therefore represents a leading process of social group maintenance [9,23]. The resulting conflict resolution, it was argued, then facilitates the transformation, in some eusocial Hymenoptera, to complex eusociality with high degrees of queen–worker size dimorphism (reproductive division of labour) and high degrees of worker polymorphism (non-reproductive division of labour) [9,24].

The current review revisits the role of conflict and conflict resolution in the major transitions. This is timely because evolutionary conflict of all kinds is increasingly recognized as exerting a profound influence on evolution (e.g. [5,25,26]; current special feature). In addition, there has been renewed interest in how conflict affects the major transitions [5,16–18,27]. In particular, in a major recent contribution, Boomsma [17] has argued for new interpretations of the processes and phenomena of the major transitions, including the role of conflict (box 2). Specifically, Boomsma [17] suggested that the role of conflict and conflict resolution in the origin of major transitions has been minimal. The current review reconsiders conflict in relation to the major transitions in light of these developments. It examines the extent to which, and manner in which, conflict and conflict resolution have influenced both the origin of major transitions and processes downstream of their origin. Throughout, it focuses on the two main fraternal major transitions, i.e. the evolution of multicellularity and the evolution of eusociality, since these are the best understood empirically and the principles they exemplify are general ones. It concludes that conflict and conflict resolution remain highly important for understanding the fraternal major transitions, including in ways not previously fully articulated.

Box 2. **Boomsma's [17] framework for the major transitions.**

In an important new book, extending his earlier contributions on the topic [12,27–29], Boomsma [17] has proposed substantial modifications to existing views of the major transitions. This box summarizes them (with significant terms emphasized), but to retain focus on the topic of the current review, it does not aim to be a comprehensive summary of Boomsma's wide-ranging work [17]:

1. Major transitions produce *organismality* or, in the case of caste-differentiated eusocial colonies, its equivalent, *superorganismality*; collectively *(super)organismality*. They therefore involve the evolution of *(super)organisms* at different levels (e.g. eukaryotic cell, multicellular organism, caste-differentiated eusocial colony).

2. Organismality represents the only seat of *group-level adaptation*, which does not occur outside (super)organisms. Organismality is distinct from individuality, though individuality may describe the later elaboration of social traits following the origin of a major transition.

3. Major transitions yielding organismality have their *origin* at the point when formerly independent units become irreversibly *somaticized*. *Somatization* means: for organelles in the eukaryotic transition, entirely enclosed by, and co-reproducing with, their host cell; and for somatic cells in the multicellular transition and workers in the eusocial transition, *irreversible* morphological commitment to their somatic/helper role, such commitment, in the case of workers in the eusocial transition, involving pre-imaginal differentiation. (In eusocial insects, such commitment also entails loss of ability to mate, although, in the eusocial Hymenoptera, non-mating worker females may still retain the ability to produce male offspring from unfertilized eggs through haplodiploidy.)

4. Social groups should be subdivided into *closed* ones (organisms or superorganisms) and *open* ones (*societies*). In this context, *closure* means genetically closed, such that no additional genes enter the social group once founded by the founding pair. *Openness* refers to the opposite, including the ability of group members to mate with partners from outside the current group. (An important consequence of such ability is, potentially, queen replacement, i.e. a prospective worker mating externally and then replacing the existing breeder.)

5. Major transitions arise phylogenetically from closed groups only and never from societies. Societies, being open, belong in a parallel *domain* but do not represent major transitions and do not provide ancestry for major transitions. Closure is hypothesized to be present before the origin of a major transition, as an exaptation, i.e. already present for independent reasons.

6. A key condition for a major transition (new level of organismality) is *lifetime commitment* of a founding pair (e.g. the female and male genomes making up the zygote in the multicellular transition; the founding paired female and male in the eusocial transition, i.e. *lifetime monogamy*).

7. In fraternal major transitions, lifetime commitment/lifetime monogamy leads to the relevant relatedness terms of Hamilton's rule (box 1) for helping by prospective helpers (cells or adult insects) being equal for the duration of the social group's life, i.e. *r*_sib_ = *r*_off_ (for multicellularity because in a clone both equal 1, and for eusociality because in a sexual family both equal 0.5). This equality (genetic indifference) holds for both the multicellular and the eusocial major transition and is the *necessary condition* for each transition.

8. As Hamilton's rule in these cases is *br*_sib_ > *cr*_off_, it reduces in both to *b*/*c* > 1. This condition, if it holds consistently for many generations, is the *sufficient condition* for the major transition. The necessary and sufficient condition together promote *unconditional altruism* and hence somatization of helper phenotypes.

9. Therefore, fraternal major transitions evolve by somatization of entire cohorts of helpers simultaneously in closed, subsocial systems. Hence organismality evolves stepwise from socially simple ancestors (that had, in the case of eusociality, parental care) and not by gradual emergence from socially complex ancestors.

In this framework [17,27], conflict and conflict resolution are concluded to have very limited roles because:

— Given the equality *r*_sib_ = *r*_off_ holds in both the multicellular and the eusocial transition (point 7 above), there is no conflict to resolve at the origin of the major transition in either the multicellular or the eusocial transition.
— As the major transition has its origin at the point when irreversible morphological commitment to helping occurs, conflict resolution after this point, especially when arising through secondary deviations from lifetime commitment/lifetime monogamy, affects only the later elaboration of social traits. An example is worker policing of male production in eusocial Hymenoptera.
— In general, within-organism conflict has limited evolutionary importance for major transitions to organismality, because, given organisms are closed systems, such conflict does not affect *resource acquisition* (e.g. investment in somatic growth) but only *resource allocation* (e.g. the distribution, after sexual maturity, of resources between male and female function), such that the essential organismality of the system is not perturbed.

While previous treatments stressed elements of this framework, such as the significance of genetic bottlenecking and a shared reproductive fate at group foundation [9,11,14,30] and organismality as the prime centre of complex adaptation [6,14,16,31,32], Boomsma's framework [17] contains several novel insights and several reworkings of existing insights endowing them with new clarity and significance, collectively representing valuable advances in understanding the major transitions. For example, the lifetime commitment concept, which identifies a correspondence between the pairwise commitment of the nuclear and organellar genomes forming the eukaryotic cell, the gametes forming a zygote and the female and male pair founding a colony, represents a powerful conceptual unification across the major transitions. Similarly, the concepts of closure and irreversible morphological commitment to helping (somatization) are key ones, with the closure concept differing from previous treatments stressing the need for social groups to exclude outside exploiters [9,10,33,34] by hypothesizing closure to be a precondition and by incorporating the exclusion of new genes that would be imported via external mating. The framework also makes novel predictions. Breaking with previous treatments, it posits that morphological queen-worker dimorphism did not evolve from behavioural queen-worker (breeder-helper) differences, since members of a behavioural worker caste might have mated externally, which would have violated the conditions of closure and unconditional altruism. Accordingly, it predicts that caste-differentiated eusocial colonies did not descend from open societies, a prediction for which there is some phylogenetic support [17].

However, elements of the framework bear further examination. A central issue is that, while it is valuable to pinpoint closure and somatization as critical elements of a major transition, exactly how each evolves is not fully specified in the framework (§§2b and 3b). The current review suggests that the necessary condition for somatization identified in the framework (point 7 above) is correct but potentially incomplete, because it does not account for inherent differences between the kin structures of clones and families, which in turn underpin key differences in the way in which conflict affects the multicellular and eusocial transitions. In particular, somatization in the eusocial transition may not be governed solely by the *r*_sib_ = *r*_off_ equality (§2b).

## 2. Conflict at the origin of the fraternal major transitions

### (a) Conflict at the origin of multicellularity

Obligate multicellularity originated through offspring cells adhering to a single founder parent cell (zygote or haploid spore) following mitotic (asexual) cell division, thereby leading to a clonal cell assemblage [35]. It therefore followed a subsocial pathway, where subsocial describes a group formed by offspring remaining associated with the parent [9]. Because such groups of cells early in this process would have been small, and so have undergone relatively few cell divisions, somatic mutations would have been negligibly rare. Hence, through the genetic identity inherent in clonality creating a complete coincidence of inclusive fitness interests between cells, potential conflict would have been absent from the incipient multicellular individual [9,35]. Therefore, as Boomsma [17] also argues, conflict and conflict resolution can have played no part in the origin of multicellularity.

However, it is worth noting that the conflict-free origin of multicellularity was possible because other conflicts had already been resolved in the precursor major transitions. These consist of: (1) intragenomic conflict introduced by the origin of genomes, resolved through the control of selfish genetic elements such as transposons; (2) intragenomic conflict introduced by the origin of sex, resolved through the control of meiotic drive genes; and (3) intergenomic conflict introduced by the origin of the eukaryotic cell (combined with sex), resolved through the control of conflict between nuclear and organellar genomes via uniparental inheritance of organelles [23,25,36,37]. Moreover, as these examples show, any major transition can, in principle, introduce a new conflict requiring resolution. The major transition leading to multicellularity did not, therefore, introduce any new conflicts at its origin. But as potentially cancer-causing somatic mutations created a risk for the organism as a whole as cell number increased following the origin of multicellularity, control of conflict between nuclear genes and such mutations became salient [9,23].

The fact that multicellular organisms originate as clonal groups of cells whereas eusocial societies originate as families in sexual species creates some important differences between these two transitions. This is the case even though, in both cases, group foundation occurs subsocially from a single founding pair (lifetime commitment in box 2). As detailed below (§2b), the clonality of incipient multicellular organisms means that not only is there no conflict as regards a cell's decision to remain with its parent cell and become a sterile somatic cell over detaching and reproducing as a unicell, because relatedness to ‘sib cells’ and relatedness to any ‘offspring cells’ both equal 1; but also there is no conflict over other decisions that the cell in principle faces. This absence of conflict, along with the preceding point regarding previously resolved conflicts, demonstrates that conflict and conflict resolution may also need to be considered by default, as the absence of conflict, and the resolution of past conflict, have important effects.

### (b) Conflict at the origin of eusociality

Caste-differentiated eusociality, i.e. in which reproductives and workers differentiate pre-imaginally (during development) to become morphologically distinct adults, originated in systems with colony founding by a single, outbred, monogamous female [28,38]. In both the diploid termites and the haplodiploid Hymenoptera, the result would be a social group consisting of a simple sexual family in which some of the female offspring (prospective workers) of the founding female (the prospective queen) can decide between rearing the reproductive offspring of the founding female, their full sibs, or leaving to produce their own offspring. (In termites, workers can also be male, but, for simplicity, from now on I refer to workers as females.) In such a family, prospective workers are genetically indifferent between rearing sibs and producing offspring [39]. This is because their relatednesses to full sibs and offspring are the same, 0.5. (Relatedness to sibs of 0.5 holds for both sexes of sib in diploids and is correct in haplodiploids taking the mean of relatedness to sisters, 0.75, and brothers, 0.25.) Therefore, Hamilton's rule (box 1) is satisfied with maximal ease in this case, as helping undergoes selection when *b*/*c* > *r*_off_/*r*_sib_, i.e. when *b*/*c* > 1, meaning that rearing sibs need be only slightly more efficient than producing offspring to undergo selection.

Boomsma [12,27,28] developed the concept of lifetime monogamy, stressing that monogamy of the founding pair is, crucially, combined in the termites and Hymenoptera with a lack of remating by the founding queen (lack of remating promiscuity) because the mating system involves the queen remaining for life with the original male partner (termites) or mating only at the beginning of adult life (Hymenoptera). This creates a lifetime commitment of the genes of the founding pair (the single queen × single male mating) that maintains the prospective workers' genetic indifference between sib-rearing and offspring-rearing for all of the queen's life. In turn, this enables the irreversible evolution of a pre-imaginally differentiating, permanent morphological worker caste [12,27,28], a process termed somatization in Boomsma's framework [17] (box 2). Building on these concepts, West *et al*. [16] highlighted that, under lifetime monogamy, prospective workers not only satisfy Hamilton's rule with maximal ease (genetic indifference), but also experience a complete coincidence of inclusive fitness interests with respect to providing help (no conflict). This is because not only does *r*_sib_ = *r*_off_ for each prospective worker, but also *r*_sib_ takes an equal value (0.5) for each such worker, implying complete evolutionary agreement between workers in the provision of help to sibs.

These insights are correct and valuable but can be fruitfully placed in a wider context. For this, it is useful to consider the evolutionary choices faced, in principle, by prospective workers in the simple family structure preceding caste-differentiated eusociality as entailing a set of three conditional decisions [cf. 40], over each of which a focal prospective worker is (by default) assumed to have control, as follows:

*Decision 1 (whether to help decision)*: As an adult, (a) remain and help rear sibs versus (b) disperse to produce one's own offspring.
*Decision 2 (caste fate decision)*: As an immature individual (larva), (a) develop as an adult worker versus (b) develop as an adult dispersing reproductive phenotype (e.g. new queen).
*Decision 3 (queen replacement decision)*: As an adult worker, (a) lose the ability to mate (with an external mate) and to replace the founding queen (queen replacement) versus (b) retain this ability.

**Figure 1.**
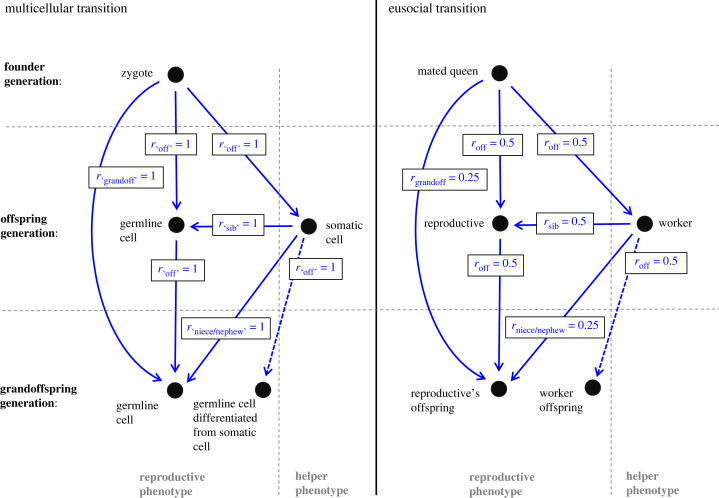
Kin structures of social groups in the fraternal major transitions. On the left: kin structure at the origin of multicellularity; on the right: kin structure at the origin of caste-differentiated eusociality (in diploids or, with modifications below, haplodiploids). The founder generation consists of a single zygote (multicellular transition) or once-mated female or queen (eusocial transition), which may produce reproductive or helper/worker phenotypes. Helper phenotypes (at the origin of the transition) potentially produce their own reproductive offspring (dashed lines). Arrows connect parties by genetic relatedness: *r*_grandoff_, relatedness to grandoffspring; *r*_niece/nephew_, relatedness to nieces or nephews; *r*_off_, relatedness to offspring; *r*_sib_, relatedness to sibs. For the multicellular transition, these values capture essential relationships but classes of relative are shown in inverted commas because cell division occurs over multiple generations of cells. For the eusocial transition in haplodiploids, in the 'whether to help' decision (§2b), *r*_sib_ = 0.5 because it is the mean of 0.75 relatedness to sisters and 0.25 relatedness to brothers, and, in the caste fate and queen replacement decisions (§2b), relevant values of *r*_niece/nephew_ and *r*_sib_ are 0.375 and 0.75, respectively.

For an irreversible morphological commitment to a worker phenotype to originate, each of these decisions has to be made in favour of the first option within it, i.e. choices 1a, 2a and 3a. The argument of the current review is that each such decision is differentially subject to potential conflict as regards the interests of the focal individual making the decision and those of the other parties within the social group. Specifically:

*For decision 1, genetic indifference is present and potential conflict is absent*: The prospective worker chooses between rearing sibs (*r*_sib_ = 0.5) or offspring (*r*_off_ = 0.5) (figure 1). So, as discussed above, there is genetic indifference for the focal individual over helping, and, because *r*_sib_ = *r*_off_ = 0.5 is true for every worker, workers are not in conflict over providing help. These equivalences are the ones highlighted by Boomsma's framework [17] as representing a crucial correspondence with the case arising in the origin of multicellularity, in which *r*_sib_ = *r*_off_ = 1 for every cell, so underpinning the conclusion in [17] that conflict plays no part in the origin of the fraternal major transitions (box 2).

*For decision 2, genetic indifference is absent and potential conflict is present*: The prospective worker, in developing as a worker and not as a reproductive, effectively chooses between allowing a sib to become reproductive in its place (*r*_niece/nephew_ = 0.25 or 0.375) or producing its own offspring (*r*_off_ = 0.5) (figure 1), removing genetic indifference. The difference from decision 1 arises because it is assumed [41] that there is an optimal timing of the onset of the production of reproductives and/or an optimal ratio of adult workers to reproductives, such that developing as a reproductive limits the choice of another female (in the simplest case, by leading to another female larva becoming a worker). Moreover, because *r*_off_ > *r*_niece/nephew_ for every developing individual, whereas the founding queen and adult workers are (within each caste) equally related to developing individuals, there is potential conflict with adult colony members over the caste fate decision [41]. This represents a new conflict introduced at the origin of eusociality. If larvae can resist being forced to develop into workers, actual conflict will ensue. However, the conflict may often fail to become actual, since in many cases it seems likely that self-determination of caste fate would be lacking because the founding queen or adult workers would have the power to control the caste fate of developing larvae [41]. Therefore, for this decision, potential conflict is present but its importance is likely to depend on biological details of the system and it may frequently not become actual and so not require resolving.

*For decision 3, genetic indifference is present and potential conflict is present*: The prospective worker, in replacing the founding queen, trades sibs (*r*_sib_ = 0.5) for offspring (*r*_off_ = 0.5) and so genetic indifference is present. However, the founding queen trades offspring (*r*_off_ = 0.5) for grandoffspring (*r*_grandoff_ = 0.25) and the other workers trade sibs (*r*_sib_ = 0.5) for nieces or nephews (*r*_niece/nephew_ = 0.25 or 0.375) (figure 1), creating potential conflict with the founding queen over whether and when to replace her and with other workers over which particular worker should be the replacement queen [42,43]. This represents another new conflict introduced at the origin of eusociality that, in principle, required resolution for the major transition to be completed.

The genetic indifference of the focal individual in decision 3 might suggest that giving up the ability to exhibit queen replacement should evolve as readily as staying within the family to rear sibs. But although *r*_sib_ = *r*_off_ = 0.5 in this case for the focal individual as in decision 1, this is of course predicated on the queen remaining present as a productive source of sibs. (The corresponding point does not apply in the multicellular transition, because the germline is a lineage of cells, so its continued presence is not reliant on a single individual surviving.) Therefore, if there is a relatively high risk of the queen losing fecundity or dying, plus a relatively high chance of successfully competing to replace her, workers might be selected to retain their mating and queen replacement abilities. Such conditions would have been particularly likely in small groups, since in small groups queens are less long-lived relative to workers and workers face relatively fewer competitors for the queen position [24,44]. Small group size is exactly the condition expected at the earliest stages of eusocial evolution [9,24,44]. Therefore, prospective workers at the origin of eusociality might be expected to have resisted somatization up to the point at which successful queen replacement became non-viable, after which actual conflict over queen replacement would have been resolved. This suggests that additional factors must become operational if somatization is to evolve (§3b).

Potential conflict in decision 2 could occur in a closed system, but conflict in decision 3 requires an open one (as the worker replacing the queen needs to have mated with an external partner). In Boomsma's framework [17], eusocial colonies are hypothesized to descend only from closed social groups and never from open ones (box 2). If correct, this would preclude queen replacement and the associated conflict occurring. However, it is not definitively established that caste-differentiated eusocial colonies evolved only from closed groups. Even if lineages with caste-differentiated eusociality did not originate within extant lineages of open societies [17], it is hard to see why the origin of eusociality would, *a priori*, exclude a stage in which the workers’ adult phenotypes, and the mating system, allowed them to exhibit queen replacement. This would have required only that adult workers be able to mate with an external partner and remain in the group. External mating by females while associated with the nest occurs widely in solitary Hymenoptera, in which males can outbreed with emerging females or females provisioning nests [45]. Likewise, external mating followed by queen replacement is frequent in non-caste-differentiated eusocial colonies [43,46–49]. Indeed, the possibility of queen replacement (choice 3b) could facilitate remaining in the nest as a helper (choice 1a), through initially providing a direct fitness benefit to supplement the indirect fitness benefit of helping [48,50]. In addition, if, given closure, somatization occurs simultaneously in entire cohorts of prospective workers (box 2), it is not clear why complete worker sterility (loss of the sperm receptacle and ovaries) seems to appear late in eusocial evolution. For example, among lineages with caste-differentiated eusociality, workers have retained sperm receptacles in bumblebees and vespine wasps [51,52] and worker loss of ovaries in ants appears relatively rare and phylogenetically patchily distributed [53].

These points imply that the hypothesis that closure is a given precondition (box 2), though it is novel and requires further investigation, may not be well supported. Alternatively, as previous treatments have suggested [e.g. 9,44], closure could be a trait evolvable in the major transition in its own right. In this view, somatization might occur by degrees, e.g. through loss of a sperm receptacle/mating ability followed by loss of ovaries, with each of these steps occurring conditional on selective conditions favourable to it [22,24]. This is because closure and somatization are not necessarily independent processes, as somatization involving the loss of mating ability would, through preventing the import of external genes, have contributed to the evolution of closure. This would make closure a consequence rather than a precondition of the transition and would also represent the evolution of closure from an open ancestor. Overall, therefore, the possibility of queen replacement may have affected the eusocial major transition in ways not captured in Boomsma's framework [17].

Finally, the existence of the three decisions described above shows that the analogy between the multicellular and eusocial transitions is not total. In organisms at the origin of multicellularity, clonality means that every pairwise relatedness value between cells is 1 (figure 1). Therefore, for the multicellular transition, no potential conflict exists in any analogue of decisions 1–3. In addition, although there is a correspondence across the two transitions between the kin structure of decision 1 (i.e. between *r*_sib_ = *r*_off_ = 1 for the multicellular transition and *r*_sib_ = *r*_off_ = 0.5 for the eusocial one), this is not the case for decisions 2 and 3. For example, a prospective somatic cell remains genetically indifferent over whether it or any other cell becomes a germline cell, but a prospective worker is not genetically indifferent over whether it or any other worker becomes a replacement reproductive (figure 1). These differences arise essentially because a clone is free of any potential conflict in a manner that a sexual family is not, even if there is lifetime commitment/lifetime monogamy. It follows that conflict and conflict resolution may, after all (box 2), have influenced the origin of the fraternal eusocial transition.

## 3. Conflict following the origin of the fraternal major transitions

### (a) Conflict following the origin of multicellularity

As discussed in §2a, there is no potential conflict between cells in a multicellular organism at the origin of multicellularity because they are all clones of one another [9,14,17]. However, as group size (cell number) increases, and given the multigenerational, branching nature of cell division underpinning growth in multicellular organisms, the potential for conflict between cells arises. This is because, after many cell divisions, somatic mutations may accumulate, degrading clonality, and such a process may cause cancers (unregulated cell division) [10,14,26]. A non-transmissible cancer and its parent body have differing inclusive fitness optima because the somatic mutations governing the cancer's growth are unrelated to genes at corresponding loci in the gametes [9]. The resulting potential conflict can of course become actual, through the growth and spread of the cancer harming its parent body and bodies evolving mechanisms that mitigate such harm. Reeve & Jeanne [54] investigated another possible consequence of somatic mutations via their concept of virtual dominance. The virtual dominant in a social group (in which any form of physical dominance is not feasible) can hold a stable reproductive monopoly because it is, by definition, the group member to whose offspring other group members have the greatest mean relatedness [54]. Any cell lineage with a reduced post-zygotic mutation rate would be a candidate for virtual dominant (here, a germline), because other cell lineages would, on average, be more related to it than they are among themselves. Reeve & Jeanne [54] therefore hypothesized that this promoted the evolution of a segregated, early-diverging germline as cell number increased [9]. If so, this would represent an increase in the degree of individuality, as it would increase the degree of reproductive division of labour [9,54]. Conflict within multicellular organisms between mutated and unmutated genes of the nuclear genome may therefore induce forms of conflict resolution with important evolutionary consequences, including for the evolution of individuality. Likewise, conflict between nuclear genes and the large array of selfish genetic elements affecting reproduction in multicellular eukaryotes [23,25,36,37] requires resolving for the multicellular organism to remain stable.

Boomsma's framework (box 2) for the major transitions proposes that conflict within closed multicellular organisms (and eusocial colonies) is not over *resource acquisition* (adaptations for acquiring resources for somatic growth) but over *resource allocation* (post-sexual maturity allocation decisions such as those between male and female function), with the result that conflict does not disrupt the essential organismality of the system [17,27]. This would support the idea that conflict and conflict resolution have minor roles in the multicellular and eusocial transitions. However, although there may be less conflict over resource acquisition precisely because parties have a higher degree of coincidence of inclusive fitness interests over somatic growth than over sex allocation, etc. [2,9], it is not clear that resource acquisition and resource allocation can be wholly separated from one another as proposed (box 2). General models of adaptation suggest that, within organisms, conflict combined with pleiotropy can lead to maladaptation even in conflict-free traits, eroding the distinction between conflict-free resource acquisition and conflict-affected resource allocation [8]. In addition, the example of non-transmissible cancers shows that, even though they evolve within the closed system of the parent body for only the duration of the parent body's lifetime, they can severely affect somatic function. More generally, lineages in which conflicts following the origin of a major transition became overwhelming and led to extinction would not, of necessity, be ones observed today. Hence one cannot necessarily deduce all evolutionary effects of conflict from the observed social organization and stability of extant lineages.

### (b) Conflict following the origin of eusociality

Multiple forms of potential conflict occur within caste-differentiated eusocial colonies, including over male parentage, caste fate and sex ratio. Many become actual, manifesting themselves in behaviours such as matricide, fighting and egg-eating [1–3,55]. Some are subject to processes of conflict resolution, for example, by social control of worker reproduction (worker policing) or caste fate [22,41,56]. The size-complexity hypothesis [9,24,57,58] proposed that, by saving costs of conflict, these processes of conflict resolution permitted increases in colony productivity and size. In turn, increasing colony size facilitated conflict resolution by leading to decreased chances of successful queen replacement and decreased powers of physical dominance, reproduction and self-determination of caste fate on the part of any one individual [9,24,44]. An important consequence was, with reproductive monopoly by physical dominance precluded, the emergence in large colonies of the queen as the virtual dominant, as workers in subsocial kin structures are more closely related to their mother's offspring than to those of any other worker and experience a coincidence of fitness interests as regards helping rear her offspring [9,54] (figure 1), so increasing the degree of reproductive division of labour. Parallel effects of large colony size selected for a greater degree of non-reproductive division of labour [9]. Overall, therefore, colony size, conflict resolution and social complexity might coevolve in a process of positive feedback, resulting, via social group transformation, in the evolution of the maximal degree of individuality and the completion of the major transition [9,18,59].

Boomsma's framework [17] for the major transitions regards such processes and phenomena as elaborations in individuality occurring downstream of the origin of the major transition itself, which is interpreted as having already occurred via the appearance, relatively quickly in evolutionary time, of a permanent morphological worker caste incapable of mating (somatization) (box 2). However, processes of positive feedback may help address a potential difficulty in Boomsma's framework, which is the condition that Hamilton's rule for the case (*b*/*c* > 1) must hold without fail for many generations to provide the directional selection necessary for the evolution of somatization (box 2, point 8). The potential difficulty is that, unqualified, this concept seems to require external ecological factors to maintain *b* > *c* for many generations, and this may be unlikely given documented effects of environmental variation on the relative magnitudes of the *b* and *c* terms in Hamilton's rule [60]. By contrast, if, as described above, conflict resolution, social complexity and greater colony size and productivity mutually reinforce one another through positive feedback, there would be an internal dynamic synergistically elevating *b* each generation. This could ensure that the sufficient condition of *b* > *c* held even in the absence of constantly favourable ecological conditions. If so, conflict resolution would remain as a factor in facilitating the evolution of the irreversible, permanent morphological worker caste (after many generations of *b* > *c*) and bringing the major transition to completion.

Recalling the proposition that conflict within closed (super)organismal systems is not over resource acquisition but over resource allocation (box 2), one also needs to consider whether this is the case in eusocial colonies. Several conflicts occurring within closed colonies of eusocial Hymenoptera suggest otherwise. For example, in sex allocation conflict, a model showed that whether this conflict is associated with single-party or mixed control of sex allocation affected colony life-history and, in particular, could reduce colony productivity [61]. In caste fate conflict, female larvae with self-determination that develop as queens thereby fail to become workers, so reducing the size of the workforce. More formally, the evolutionarily stable proportion of queens was higher for individual female larvae than for adult workers [62,63], so conflict in this case again affects colony growth and hence resource acquisition. Lastly, in conflict over male parentage, the anticipation of direct fitness through laying male eggs can affect workers' rates of performance of worker-like behaviours when the queen is alive [64,65], and workers may even abandon the natal colony entirely to produce males in other colonies [66], both of which phenomena potentially affect resource acquisition. Conflict over male parentage can even lead to worker matricide [67–69], an outcome whose analogue in multicellular organisms (the soma destroying the germline prematurely against the germline's interests) does not exist because of the dissimilarities in kin structure across the two transitions previously highlighted (figure 1). In summary, conflict within the closed system of caste-differentiated eusocial colonies can disrupt resource acquisition in ways that are predictable, via inclusive fitness theory, from these colonies’ sexual family kin structure. Although in a closed system the same genes remain present, bundling them into different packages through sexual reproduction creates colony members with divergent inclusive fitness interests. By implication, resolving these conflicts, even partially, would mitigate such effects, and so could affect the organismality and superorganism-level adaptations of the colony.

## 4. Discussion

The conclusion drawn from this review is that conflict and conflict resolution are likely to have been operative factors in the post-origin stages of the transition to multicellularity and in both the origin and post-origin stages of the transition to eusociality. Conflict was absent at the origin of multicellularity because clonality within a small subsocial group of cells leads to a complete coincidence of inclusive fitness interests. Overall, therefore, conflict remains highly important for understanding the fraternal major transitions.

This conclusion clearly raises other important considerations, one of which concerns the concepts of individuality and organismality. Individuality, as earlier defined (§1), is a broader concept and essentially includes organismality as its most extreme expression. From first-principles-based inclusive fitness reasoning, individuality, including that shown by conventional organisms and eusocial colonies, evolves in a major transition as a function of the coincidence of inclusive fitness interests of the constituent parties [7,9,14]. In Boomsma's framework [17,27], organismality (including the superorganismality of caste-differentiated eusocial colonies) evolves for essentially the same reason but is a more tightly defined quality. In particular, it is taken to be the only product of a major transition (i.e. entities that are not organisms in the sense of the framework are not products of a major transition), to occur only within closed groups and to be the only seat of group-level adaptations (box 2).

Some cases, however, seem to blur the edges of this characterization of organismality. There are social groups that are non-superorganismal (by the definition in box 2) but exhibit a high degree of individuality and candidate group-level adaptations. Examples include colonies of the naked mole-rat (*Heterocephalus glaber*) and the epiponine wasps (Epiponini), both of which are open and lack irreversible morphological workers. In both systems, there is a complete interdependence of reproductive and non-reproductive phenotypes in that colony life is obligate and neither phenotype can generate progeny without the other. Therefore, even though the system is not closed and workers are not somaticized, there is a degree of individuality very similar to an organismal one. In addition, in both systems, there are variously quite sophisticated communal structures (tunnel system, nest), forager recruitment behaviours and communal nest defence behaviours [33,70–73]. These collective phenotypes represent candidate group-level adaptations in the sense that they benefit the colony as a whole and, specifically, that improvements to them, just as in closed superorganisms, could undergo selection solely via indirect fitness benefits to non-reproductive workers mediated through increasing queen reproduction. Therefore, it is questionable whether group-level adaptations are restricted to closed superorganisms in the sense of box 2. If so, the evolution of a high degree of individuality remains a viable end-point for a major transition.

A related consideration invokes a broader view of what constitutes adaptation in entities undergoing major transitions. This view holds that adaptation can reside at several levels within an individual. For example, in a conventional multicellular organism, there are maladaptations at the higher level (organism level) that can be explained as adaptations at the lower level (within-organism level) of the selfish genetic elements causing them, such as meiotic drive genes or, in plants, mitochondrial genes causing cytoplasmic male sterility [74]. Adaptation still serves to maximize the inclusive fitness of the gene or genes responsible for the adaptive phenotype [6,17], and, when many genes share a coincidence of inclusive fitness interests in this respect, the result is still complex adaptation at the group (higher) level [7]. Nonetheless, adaptation does not reside just at the higher level, and, at that level, remains conditional on attenuation, through conflict resolution, of the within-individual conflict that is maladaptive at the higher level [6,7,9,26].

Reflecting key differences between sexual families and clones discussed earlier (§2b), it is also worth recollecting that a sexual family, including a eusocial colony, unlike a multicellular organism (as represented by its autosomal chromosomes), has no single inclusive fitness for adaptation to maximize. This is because the colony's inclusive fitness cannot be defined [9]. Only the inclusive fitnesses of the constituent kin groups within it (queen, workers, self) can be defined [e.g. 75], which may differ (so causing potential conflict) but will generate a colony-level adaptive phenotype whenever they coincide. This creates another reason for regarding complex adaptations as the product of a coincidence of inclusive fitness interests with respect to a given trait and context [7], and therefore not *a priori* restricted to occurring in closed (super)organisms alone.

Taking the product of major transitions to be the broader quality of individuality, as opposed to (super)organismality in the sense of box 2, also allows the major transitions framework, coupled with inclusive fitness theory, to address non-standard organisms that may, nonetheless, exhibit high degrees of individuality [76]. Lastly, although the degree of individuality can vary, the individuality concept does not imply that thresholds in the major transitions are unimportant, the chief one being irreversible morphological commitment to a helper phenotype as identified by Boomsma's framework [12,17,18]. But an outcome that is stepwise when viewed in evolutionary time may still occur in distinct events [18]. Moreover, given that the processes that bring a system across the threshold may resemble those that subsequently continue to increase the degree of individuality, it seems legitimate to view the major transition as being completed when the degree of individuality has attained its maximal value.

## Data Availability

This article has no additional data.
